# Luminous vs. nocturnal canopy: a comparative qualitative study of two VR horticultural scenes for relaxation

**DOI:** 10.3389/fpsyg.2026.1932971

**Published:** 2026-10-05

**Authors:** Fan Zhang, Chenyuan Gao, Lu Huang

**Affiliations:** School of Environmental Art, Hubei Institute of Fine Arts, Wuhan, China

**Keywords:** virtual reality, horticultural environments, environmental psychology, qualitative study, user preference, field notes

## Abstract

**Introduction:**

Virtual reality (VR) horticultural environments are an emerging area, yet little is known about how environmental visual tonevisual tone, as one dimension of the overall environment, may be associated with influences user experience in therapeutic contexts.

**Methods:**

We designed two VR horticultural scenes with different visual tones, and collected field notes from participants who experienced both scenes during an 11-day public exhibition. Quantitative preference and relaxation scores were also collected.

**Results:**

Thematic analysis of field notes (*N* = 14) revealedgenerated four main themes: (1) emotional and sensory responses to the two scenes with Luminous Canopy associated with vitality and hope while Nocturnal Canopy associated with calmness, introspection, and tranquility; (2) plant familiarity and recognition, where familiar plants were associated with comfort while unfamiliar plants were associated with curiosity; (3) a safety–exploration tension across scenes; and (4) contextual fit and situational preference, with preference varying by mood and context. Quantitative results showed that 64.3% of participants preferred the Luminous Canopy.

**Discussion:**

Participants’ responses suggest that personalized VR therapeutic environments may be beneficial for future therapeutic environment design. Participants’ diverse preferences suggest that offering multiple scene options may better accommodate different user needs.

## 1 Introduction

### 1.1 Horticultural environments and psychological wellbeing

Horticultural environments, as a complementary approach, have been associated with mental health benefits through human-plant interactions (Soga et al., 2017). Previous studies have shown that horticultural activities have been associated with improved mood, reduced perceived stress, and enhanced social connection (Clatworthy et al., 2013; Gonzalez et al., 2010). Attention Restoration Theory (Kaplan, 1995) suggests that natural environments are associated with the restoration of directed attention through the mechanism of soft fascination, which may help explain the potential benefits of such environments. Beyond these general benefits, emerging evidence suggests that specific visual features of plant environments—such as color, brightness, and spatial arrangement—may be associated with emotional responses, although these factors remain underexplored in the context of these environments.

### 1.2 Virtual reality as a medium for therapeutic environments

Virtual reality offers new possibilities for therapeutic environments. It is an interactive 3D environment where users share a virtual space with a realistic sense of depth and immersion (Bernhard and Shamri Zeevi, 2025). VR-based applications combine traditional elements such as line, form, and color with digital tools, allowing unrealistic features not bound by physical laws (Hacmun et al., 2018). Users can remove the headset when difficult emotions arise, providing a sense of control.

Evidence shows VR-based therapeutic environments are associated with reduced anxiety, improved emotional processing, and enhanced engagement (Hacmun et al., 2018). VR environments foster engagement and symbolic expression (Haeyen et al., 2021). Systematic reviews highlight the need for accessible VR applications (Best et al., 2022). Recent research has explored the design of horticultural therapy content in VR environments, demonstrating that users can achieve psychological stability through plant management activities (Kim et al., 2024). VR forest environments have also been shown to improve mood and restorative outcomes in older adults (Yuan et al., 2022). While some of these studies originated within the context of art therapy, they provide relevant evidence for VR-based therapeutic environments more broadly, particularly regarding presence, immersion, and user engagement. However, how visual tone affects user experience in VR horticultural environments remains unclear. Most studies ask whether VR environments work, not what environment suits whom. Bernhard and Shamri Zeevi (2025) found that VR facilitated emotional communication in mother-daughter dyads, but did not examine how visual factors like plant color or lighting shape experience, a gap this study addresses. While previous studies have focused on the general effectiveness of VR environments, the present study employed a dual-scene comparative design to examine how variations in environmental visual tone are associated with user experience.

### 1.3 Environmental psychology: the role of visual tone

Research in environmental psychology suggests that visual environmental tone is associated with individual emotions and psychological states. In this study, visual tone refers to the overall visual atmosphere created by the combined use of color palette, brightness level, and lighting conditions within a virtual environment. Warm-toned or bright environments are generally associated with positive emotions and vitality (Elliot and Maier, 2014), while cool-toned or dark environments are associated with calmness and introspection (Li et al., 2020). Beyond overall tone, specific visual features such as plant color and lighting intensity have been associated with emotional responses and perceived restoration (Ai et al., 2025; Hirakawa et al., 2025). Plant color combinations have also been associated with stronger relaxation effects than single-color plant communities, with green and yellow combinations producing the most pronounced relaxation responses (Zheng et al., 2024). Exposure to green plant colors has been associated with increased relaxation and internal concentration (Jang et al., 2019). Immersive virtual nature environments have also been associated with reduced anxiety and negative affect compared to urban environments (Barth et al., 2025). VR-based nature environments have also been associated with enhanced positive affect and reduced perceived stress (Schuil et al., 2026). VR forest mindfulness interventions have been associated with sustained mood recovery effects (Jiang et al., 2025). However, in the field of VR therapeutic environment research, how environmental tone may be associated with user experience remains unclear. Most existing studies focus on whether VR environments work, with less attention paid to what kind of VR environment works for whom, the question of personalization.

### 1.4 Research aims and questions

This study aims to fill this gap by designing two VR horticultural scenes with different visual tones and exploring how two different environmental configurations are associated with user experience. The specific research questions are as follows:

RQ1: What emotional experiences are associated with Luminous Canopy and Nocturnal Canopy respectively?
RQ2: How is user preference distributed between the two scenes, and what factors are associated with preference?
RQ3: What user states and needs are associated with each scene?

The two VR scenes were developed as part of a project titled *Grounded*, which explores the potential applications of immersive horticultural environments through comparative scene design.

## 2 System design: two VR horticultural scenes

### 2.1 Shared design philosophy

Both scenes are designed from a recumbent perspective, with plants suspended downward from the ceiling to form a canopy-like visual layer above the user. The two scenes share the same design goals of supporting emotional relaxation and restorative experiences through immersive horticultural experiences, and both incorporate a variety of plants with distinct forms and colors. In both scenes, users lie in bed and observe the suspended plants from below, with interaction facilitated through simple controller-based input. Each complete experience session lasts approximately 3 to 5 min. The entry interface of the VR experience is shown in Figure 1a.

**Figure 1:**
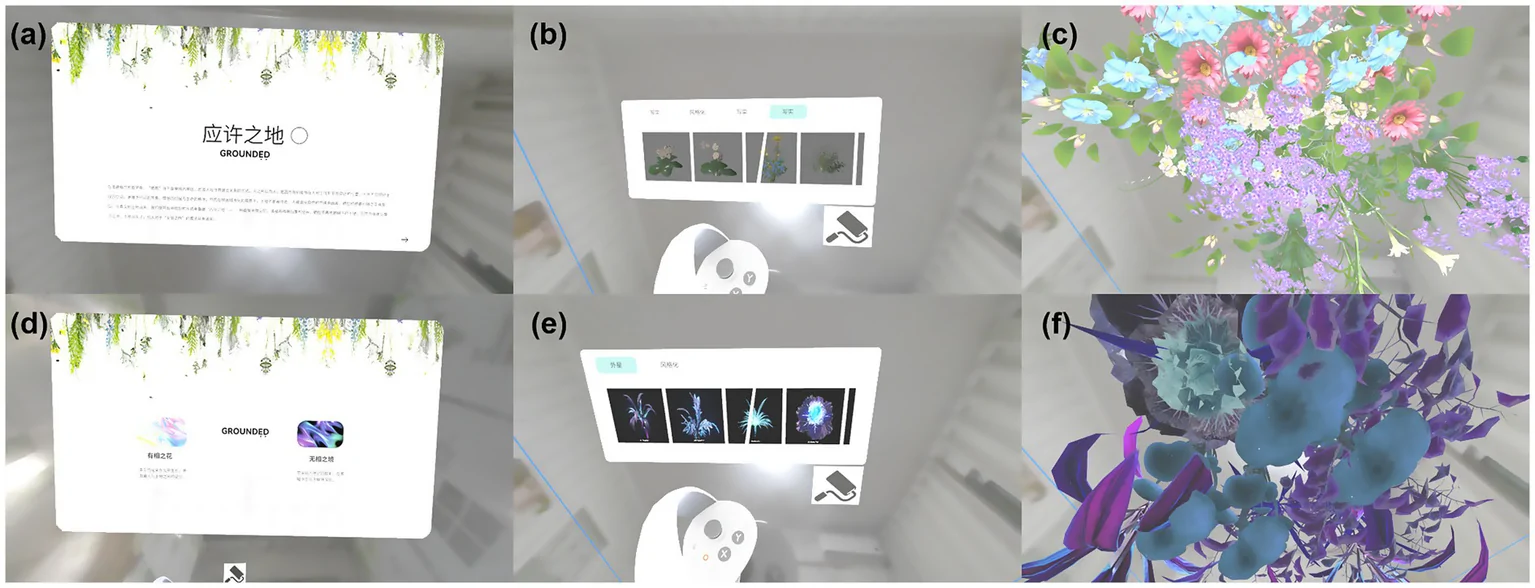
**(a)** Entry interface of the Grounded VR experience. **(b)** Plant selection interface for Luminous Canopy. **(c)** Luminous Canopy scene screenshot. **(d)** Scene selection interface showing the two scene options. **(e)** Plant selection interface for Nocturnal Canopy. **(f)** Nocturnal Canopy scene screenshot.

### 2.2 Scene 1: luminous canopy

Luminous Canopy features flowers suspended from the ceiling, including daisies, lavender, dandelions, and other blossoms in pink, light blue, and light yellow. Daisies and dandelions are widely recognized, while lavender is commonly associated with calmness. The scene uses a warm palette of yellow, green, light purple, and white with high lightness, illuminated by bright daylight. The auditory design includes bird calls from the left, stream sounds from below, and bright background music (piano or acoustic guitar in major key). The design draws on color psychology, which associates flowers with positive emotions (Elliot and Maier, 2014), attention restoration theory, which suggests that natural forms can support attentional recovery (Kaplan, 1995), and spatial audio technology, which is used to support presence in virtual environments (Hacmun et al., 2018) (Figures 1b,c).

### 2.3 Scene 2: nocturnal canopy

Participants selected between the two scenes through a scene selection interface (Figure 1d). Nocturnal Canopy features plants suspended from the ceiling, including glowing mushrooms, blue roses, dark orchids, and other deep-hued foliage. These plants present blurred, dissolving forms in low light; glowing mushrooms are surreal, blue roses are not easily found in nature, and dark orchids are deep in color. The scene uses a dark palette of deep green, purple-brown, dark blue, and cyan/green fluorescence, with moonlight, starlight, and self-illumination from mushrooms. The auditory design features 360-degree surround sound of night insects, distant wind chimes, and low ambient music (double bass or synthesizer pads, minor key). This design draws on research associating low-light environments with introspection (Li et al., 2020), and on VR’s unreal characteristics, which allow the creation of plant forms unavailable in physical reality (Hacmun et al., 2018) (Figures 1e,f).

### 2.4 Sensory comparison of the two scenes

Both scenes use spatial audio positioning with directional sound sources and surround effects. Luminous Canopy uses directional sound sources originating from the left and from below. Nocturnal Canopy uses a surround sound field, with insect humming around the ears and wind chimes at a distance. The two scenes therefore differ in multiple dimensions, including plant type, color palette, lighting, and sound design; the study compares these two overall configurations rather than isolating visual tone as a single variable.

### 2.5 Technical implementation

The VR scenes were developed using Unity 3D and deployed on Pico 4 VR head-mounted displays. Interaction was facilitated through Pico 4 handheld controllers with 6DoF tracking, allowing users to observe and interact with plants suspended from the ceiling while lying in bed. The Pico 4 device was selected for its all-in-one design, which requires no external PC connection and is suitable for public exhibition settings.

To enhance the sense of privacy and enclosure during the experience, the bed was surrounded on all sides by soft fabric drapes, forming a semi-enclosed space. The curtain enclosure was designed to limit visual distractions. Additionally, an external display was positioned outside the curtain area, mirroring the VR headset view in real time. This allowed researchers to monitor participant experience and also enabled waiting visitors to observe the VR content.

The public exhibition ran from May 28 to June 10, 2026, at the Wujie Art Space, Dazhi Wujie, Wuhan, China. The first two days (May 28–29) were dedicated to setting up the exhibition, and the final day (June 10) was reserved for dismantling. Formal testing was therefore conducted from May 30 to June 9, 2026 (extended by one day beyond the ethics-approved period of May 30 – June 8, 2026) (Figure 2).

**Figure 2:**
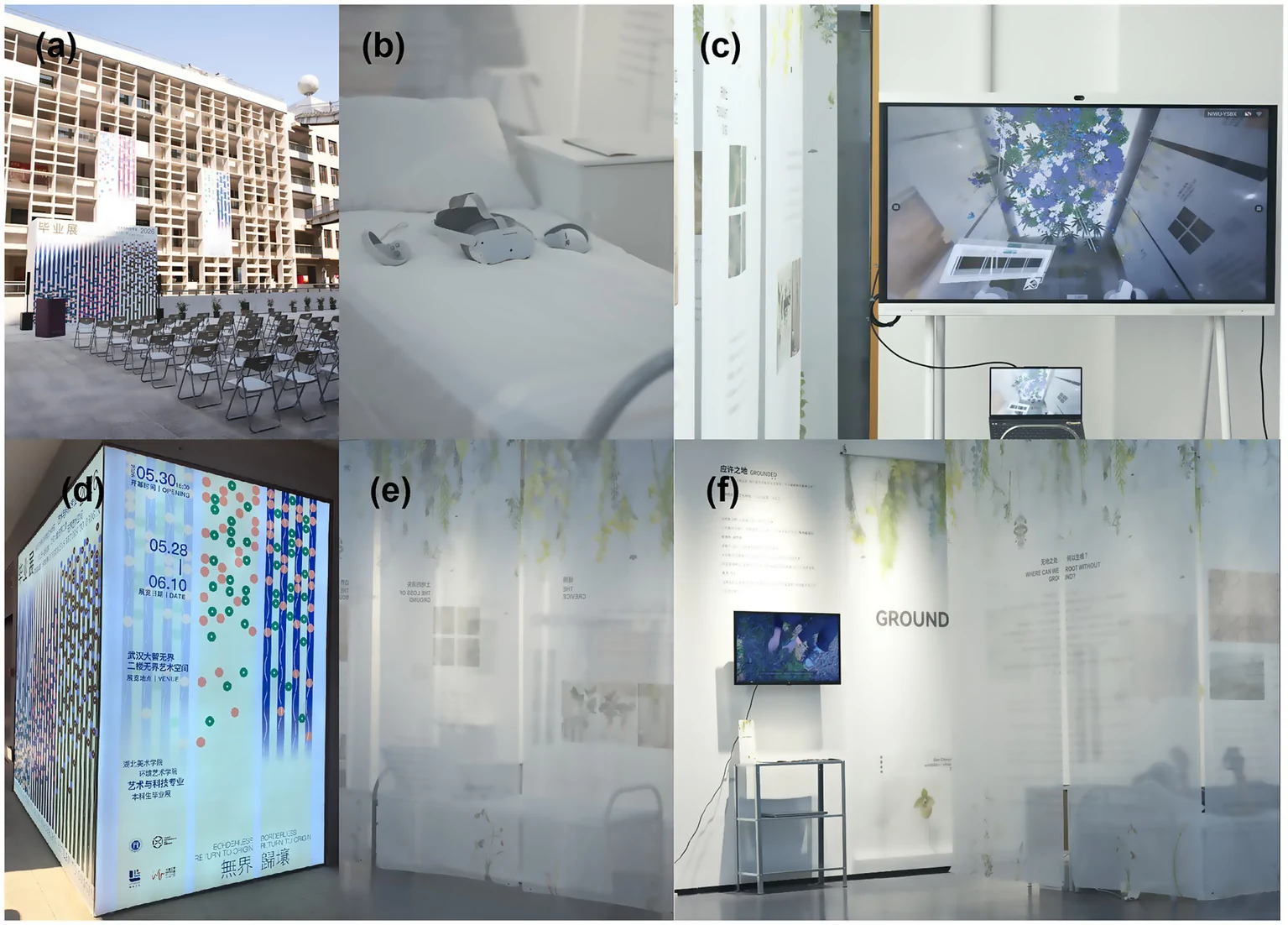
**(a)** Exhibition venue at the Wujie Art Space, Wuhan. **(b)** Experimental setup showing the bed and Pico 4 headset. **(c)** External display mirroring the VR scene in real time. **(d)** Exhibition poster on the wall showing dates and location. **(e)** Semi-enclosed space formed by the curtain drapes. **(f)** Exhibition space showing the Grounded installation and printed text on the curtains.

## 3 Methods

### 3.1 Study design

This study employs a within-subjects design, where each participant experiences both VR scenes (Luminous Canopy and Nocturnal Canopy) in sequence to control for variability across participants in experience evaluation. Presentation order was counterbalanced to control for order effects: half of the participants experienced Luminous Canopy first followed by Nocturnal Canopy, while the other half experienced the reverse order. The study is an exploratory mixed-method study, with qualitative data as the primary focus and quantitative data as Supplementary material.

### 3.2 Participants

Participants were recruited on a walk-in basis during an 11-day public exhibition (May 30 – June 9, 2026) held at the Wujie Art Space, Dazhi Wujie, Wuhan, China. Inclusion criteria were: (1) no severe visual or auditory impairments; and (2) voluntary participation with signed informed consent. A total of 14 participants were recruited (6 males, 8 females), ranging in age from 6 to 58 years (M = 29.6, SD = 14.3). Four participants had prior VR experience, while ten had none. Regarding daily nature contact, three participants reported rare contact, four reported monthly contact, five reported weekly contact, and two reported daily contact. For daily time preference, eight participants preferred daytime, four preferred nighttime, and two expressed no clear preference. For the minor participant (6 years old), written informed consent was obtained from the legal guardian prior to the VR experience, and the guardian accompanied the participant throughout the testing session. Given the wide age range, we note that age-related differences in plant familiarity, VR experience, and emotional expression may have been associated with variations in participant responses; however, the present exploratory design did not include sufficient statistical power to test age effects, and this is acknowledged as a limitation in Section 5.4. Participant characteristics are summarized in Table 1.

**Table 1:** Participant characteristics (*N* = 14).

Characteristic	Value
Total number (*N*)	14
Gender (male/female)	6/8
Age (mean ± SD, range)	29.6 ± 14.3 (6–58)
Previous VR experience (yes/no)	4/10
Frequency of daily nature contact (rare/monthly/weekly/daily)	3/4 / 5/2
Daily time preference (day/night/no preference)	8/4 / 2

### 3.3 Procedure

Throughout the experience, participants lay in bed wearing a Pico 4 headset and used handheld controllers to observe and interact with each scene. Participants first completed a brief pre-experience questionnaire covering demographics, prior VR use, and daily nature preferences. They then experienced the two VR scenes in counterbalanced order, with each session lasting 3 to 5 min. After each scene, participants rated their relaxation level on a 1-to-10 scale. A 2-min rest interval was provided between scenes to minimize cybersickness and fatigue. Following both experiences, participants indicated their scene preference and rated preference intensity (1–10). The researcher documented participants’ spontaneous verbal feedback as field notes. The researcher recorded one or two representative phrases per scene, including emotional responses, reasons for preference, and contextual fit.

### 3.4 Data collection

Quantitative measurement tools: Relaxation levels were measured using a single-item rating scale (1 = not relaxed at all, 10 = extremely relaxed) immediately after each scene. Scene preference was measured by asking participants which scene they preferred (Luminous Canopy/Nocturnal Canopy/no preference), and preference intensity was rated on a 1-to-10 scale (1 = slight preference, 10 = strong preference).

Qualitative tool: Field notes were recorded by the researcher on standardized forms during and immediately after each session. Each participant’s record contained one or two representative phrases per scene, reflecting the participant’s own verbal expressions. Verbatim phrases were recorded whenever possible, using quotation marks. These field notes served as the primary qualitative data source for thematic analysis. Field notes are a recognized data collection method in qualitative research, particularly suitable for exploratory studies with small samples where immediate post-experience feedback is documented by the researcher rather than audio-recorded (Mulhall, 2003; Phillippi and Lauderdale, 2018). To minimize interpretive bias, handwritten notes prioritized participants’ own wording, and the consolidated record retained these verbatim phrases. A reflexivity statement is provided in Section 3.5. The questionnaire template (Appendix A) and the field notes (Appendix B) are provided in the Supplementary material.

### 3.5 Data analysis

Quantitative data were analyzed descriptively using Microsoft Excel. Means and standard deviations were calculated for relaxation ratings of each scene, and the distribution of scene preference and preference intensity was summarized. Given the small sample size and exploratory nature of the study, no inferential statistical tests were performed, and the quantitative results are reported as descriptive patterns.

Qualitative data were analyzed using Braun and Clarke's (2006) six-step thematic analysis. The researcher first read all field notes repeatedly to become familiar with the data. Initial codes were then generated by labeling meaningful segments of participants’ verbal expressions (e.g., references to familiarity, novelty, safety, or emotional response). These codes were grouped into candidate themes based on shared meaning, and the themes were reviewed against the original notes to check whether they reflected participants’ responses. Themes were defined and named, and representative verbatim quotes were selected for each theme. Coding was conducted manually in Microsoft Excel. The themes were inductively derived from participants’ own expressions rather than from a predefined framework. To enhance trustworthiness, several strategies were implemented: (1) peer debriefing with a colleague not involved in data collection; (2) maintaining an audit trail of coding decisions; (3) review of preliminary themes by a co-author who was not involved in data collection, to check whether they reflected participants’ original expressions; and (4) retaining verbatim phrases in the consolidated record. We also acknowledge that the researcher who recorded the field notes also conducted the analysis, which may introduce interpretive or selection bias. This limitation is addressed in Section 5.4. Field notes were adopted as the primary qualitative data source due to the exploratory, exhibition-based nature of the study.

### 3.6 Ethical statement

This study was reviewed and approved by the Academic Committee of the School of Environmental Art, Hubei Institute of Fine Arts (Review No. SEA2602). All participants provided written informed consent. For the minor participant (6 years old), written informed consent was obtained from the legal guardian prior to the VR experience, and the guardian accompanied the participant throughout the testing session. The ethical review document (Appendix C) and the informed consent form template (Appendix D) are provided in the Supplementary material.

## 4 Results

### 4.1 Quantitative results

Relaxation ratings were summarized descriptively for each scene. The mean relaxation score for Luminous Canopy was M = 7.36 (SD = 1.28), while the mean score for Nocturnal Canopy was M = 6.64 (SD = 1.28). Both scenes had a similar spread of ratings, with scores ranging from 5 to 9 (Figure 3a).

**Figure 3:**
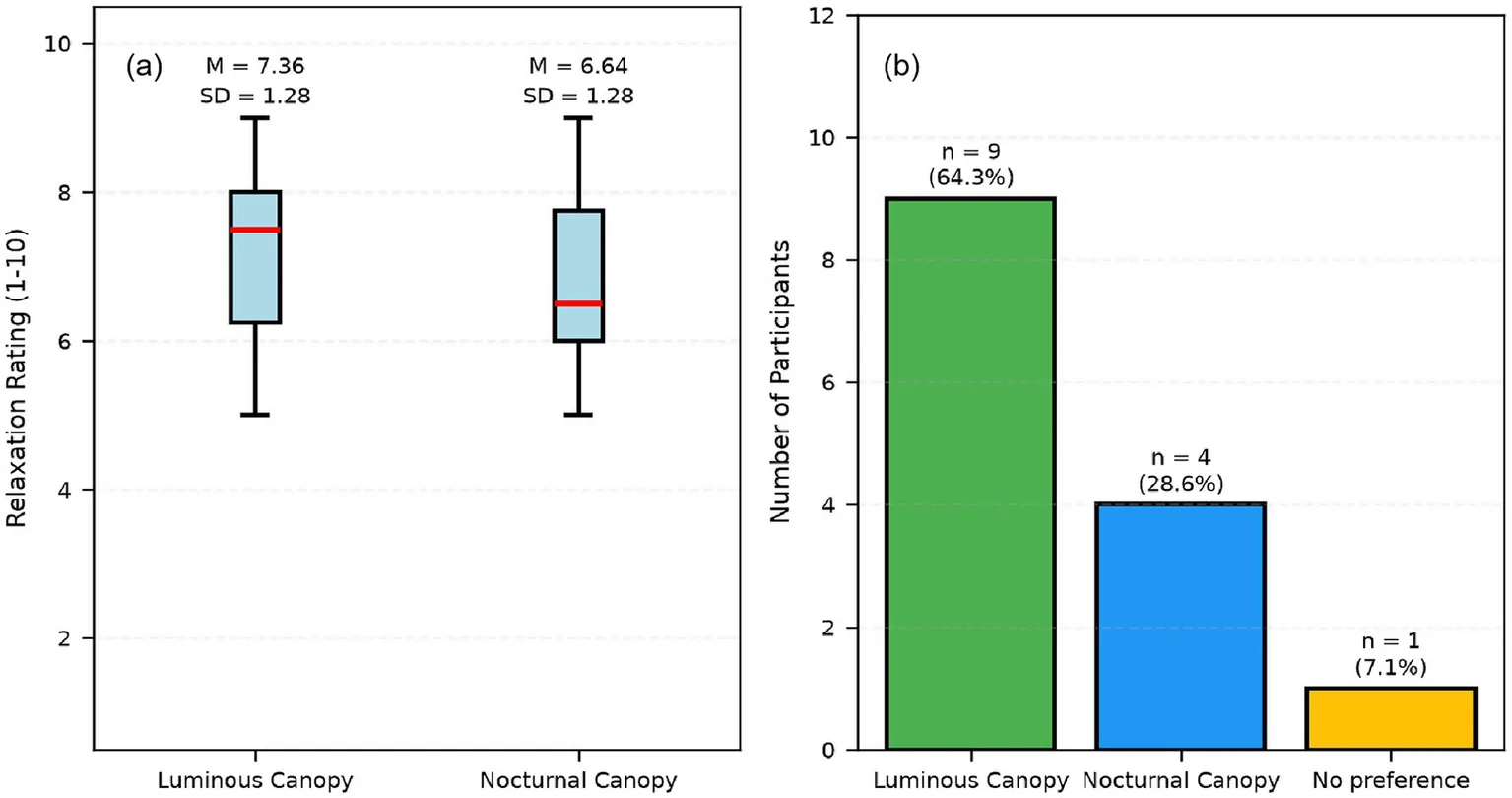
**(a)** Boxplot of relaxation ratings for both scenes. **(b)** Bar chart of scene preference distribution.

Regarding scene preference, 9 participants (64.3%) preferred Luminous Canopy, 4 participants (28.6%) preferred Nocturnal Canopy, and 1 participant (7.1%) expressed no clear preference (Figure 3b). Among those who expressed a preference, the mean preference intensity was M = 8.2 (SD = 1.3) for Luminous Canopy preferrers and M = 7.0 (SD = 1.6) for Nocturnal Canopy preferrers.

### 4.2 Qualitative results

Thematic analysis identified four interconnected themes that together describe how participants experienced and evaluated the two scenes. Theme 1 describes the emotional responses associated with each scene; Theme 2 describes differences in plant familiarity and recognition; Theme 3 describes the safety–exploration associations reported by participants; and Theme 4 describes how preference varied with context and mood. These themes are presented as sequential layers of the same experiential process rather than as independent categories.

Theme 1: Emotional and Sensory Responses to the Two Scenes. Participants consistently reported qualitatively different emotional experiences between the two scenes. Luminous Canopy was associated with positive emotions, vitality, and hope. One participant noted that seeing daisies and dandelions reminded her of childhood, while another described lavender’s purple as calming. The left-side bird calls were frequently mentioned as immediately uplifting, with one participant feeling as if birds were beside her. In contrast, Nocturnal Canopy was associated with calmness, introspection, and tranquility. Participants described glowing mushrooms as peaceful precisely because they are impossible in reality, blue roses as dreamlike, and dark orchids as associated with stillness. Distant wind chimes created a sense of vast space.

Theme 2: Plant Familiarity and Recognition. Participants showed marked differences in plant recognition. Plants in Luminous Canopy were almost universally recognized, and were associated with comfort and security. One participant explained that recognizable plants made the environment feel familiar and reassuring. In contrast, plants in Nocturnal Canopy were often described as never seen before or dreamlike. Some expressed curiosity about unfamiliar plants, while others felt slightly nervous around overly strange flora, suggesting a tension between novelty and familiarity.

Theme 3: Safety and Exploration. Luminous Canopy was consistently linked to familiarity and safety. Nocturnal Canopy was associated with novelty and exploration. One participant noted initial discomfort but subsequent peacefulness, while another described the darkness as mysterious and expressed a desire to explore further.

Theme 4: Contextual Fit and Situational Preference. Participants emphasized that scene preference was situational rather than fixed, depending on current mood and context. Participants who reported feeling tired or overwhelmed preferred Nocturnal Canopy as a quiet retreat, while those who reported being in a positive mood favored Luminous Canopy for its brightness. Participants who reported a preference for nighttime favored Nocturnal Canopy, while those who reported preferring mornings favored Luminous Canopy. Several noted that preference varied by mood, wishing both scenes were available.

## 5 Discussion

### 5.1 Summary of main findings

This study compared Luminous Canopy and Nocturnal Canopy in VR horticultural scenes. The two scenes were associated with differentiated emotional response patterns: Luminous Canopy was associated with vitality and hope, while Nocturnal Canopy was associated with calmness and introspection. Participants’ responses highlighted plant familiarity and recognition: familiar plants were associated with comfort and security, while unfamiliar plants were associated with curiosity. The two scenes also differed in their spatial audio design: Luminous Canopy used directional sound sources, while Nocturnal Canopy used a surround sound field. User preference varied across participants, with 64.3% preferring Luminous Canopy and 28.6% preferring Nocturnal Canopy. Participants described their preference as shaped by a tension between safety and exploration, as well as contextual factors such as current mood and reported daily preferences.

### 5.2 Integration with previous research

The findings align with Hacmun et al.'s (2018) principles for VR therapeutic environments in horticultural scenes, particularly unreal characteristics through surreal plants like glowing mushrooms and blue roses. Spatial audio was also incorporated as a design element. Participants reported feeling greater freedom in VR than real gardens. The findings are consistent with color psychology (Elliot and Maier, 2014) and attention restoration theory (Kaplan, 1995) in VR environments, and suggest that plant familiarity may be a relevant dimension for future research. Li et al.'s (2020) finding of an inverted U-shaped relationship between brightness and stress recovery supports personalized environments avoiding extremes. Theme 4 suggests that personalized VR horticultural experiences may benefit from diverse scene options rather than one-size-fits-all approaches.

Unlike most VR therapeutic environment studies using seated or standing postures, this study introduced a recumbent perspective with ceiling-suspended visuals, a format that may be relevant to users who prefer or require a lying posture. It also examines audio design by comparing directional vs. surround sound strategies and their associations with exploration and introspection. Furthermore, scene preference varied with context and mood rather than remaining fixed, suggesting that personalized VR environments may require dynamic adaptation to users’ momentary emotional states. These features point to postural, auditory, and dynamic personalization in addition to content design.

### 5.3 Design implications

For participants who reported seeking familiarity and safety, Luminous Canopy may be more suitable, with recognizable flowers and directional sounds (bird calls, streams). For participants who reported seeking novelty and introspection, Nocturnal Canopy features unfamiliar plants and surround sound (insects, distant wind chimes). First-time users may prefer Luminous Canopy’s familiar plants and soundscape, while participants who reported seeking novelty may prefer Nocturnal Canopy’s unfamiliar plants and surround sound field.

Two design considerations are suggested by participants’ responses: plant selection may be guided by recognizability and familiarity; sound design may be associated with spatial experience through directional sources, distance cues, and surround effects. Participants’ diverse preferences suggest that VR horticultural environments may need to consider offering multiple scene options rather than a single fixed environment.

### 5.4 Limitations

Several limitations should be noted. The small sample (*N* = 14) from a short-term exhibition limits generalizability to broader populations and long-term contexts. The wide age range (6–58 years) introduces additional heterogeneity in plant familiarity, VR experience, and emotional expression, which may have been associated with variations in participant responses; the exploratory design did not include sufficient statistical power to test age effects. The single-session design precludes assessment of cumulative effects. The two scenes differed across multiple dimensions—plant type, color palette, lighting, and sound design—so the observed differences cannot be attributed to visual tone alone; the study compared two overall environmental configurations rather than isolating a single variable. Relaxation was measured with a single-item scale immediately after each scene. This brief measure captures perceived relaxation at a single moment and cannot capture the multidimensional nature of stress or long-term psychological outcomes; future research should employ validated instruments (e.g., STAI, PSS) and physiological indicators (e.g., heart rate variability). Given the small sample size, no inferential statistical tests were performed, and the quantitative results are reported as descriptive patterns only. Qualitative data from field notes rather than audio-recorded interviews may not fully capture verbatim expressions, with potential researcher bias in selective documentation. The researcher who recorded the field notes also conducted the thematic analysis, which may have introduced interpretive or selection bias; this was partially managed through peer debriefing, audit trails, and review of preliminary themes by a co-author not involved in data collection. All participants were from a Chinese cultural context; meanings and preferences associated with plant colors and VR-based environments may differ cross-culturally, requiring future validation. Despite these limitations, the study provides preliminary insights into personalized VR horticultural environment design and offers a foundation for future research with rigorous controls and broader samples.

### 5.5 Future directions

Future research could extend the present findings in several directions. First, expanding the sample size and including individuals with anxiety or depression would enhance the generalizability of the findings and help examine the potential benefits of VR horticultural environments for those in need of mental health support. Second, adopting a longitudinal design would allow for tracking the long-term effects of repeated exposure to VR scenes, addressing a key gap in understanding cumulative effects over time. Third, incorporating physiological measures such as heart rate variability and electrodermal activity would provide more objective and fine-grained evidence of relaxation and emotional regulation beyond self-report data. Fourth, exploring additional scene dimensions, including plant type, dynamic versus static environments, and representational versus abstract styles, would further enrich the design space for personalized VR environments. Fifth, developing personalized scene recommendation algorithms based on user characteristics—such as daily nature preferences or current emotional states—could facilitate real-time adaptation and support more tailored user experiences. Collectively, these directions may contribute to both the theoretical understanding and practical application of VR horticultural environments.

## 6 Conclusion

This study designed and compared two VR horticultural scenes, Luminous Canopy (daisies, lavender, dandelions) and Nocturnal Canopy (glowing mushrooms, blue roses, dark orchids), and collected quantitative and qualitative data from a small sample of users through an 11-day public exhibition. Participants described distinct emotional response patterns associated with the two scenes, with Luminous Canopy associated with vitality and hope, and Nocturnal Canopy associated with calmness and introspection. User preference varied across participants, associated with current mood and reported daily preferences. In addition, participants’ responses highlighted plant recognizability (familiar versus unfamiliar) and spatial audio positioning (directional sound sources versus surround sound fields) as dimensions that were associated with user experience. Participants’ diverse preferences suggest that VR horticultural environments may benefit from offering diverse scene options rather than a one-size-fits-all approach to accommodate users’ differing needs. This study provides preliminary insights into plant and sound design in VR horticultural environments. Future research could explore more personalized VR experiences based on these findings.

## Data Availability

The original contributions presented in the study are included in the article/Supplementary material, further inquiries can be directed to the corresponding author.
