# Supplementary material for: Luminous vs. nocturnal canopy: a comparative qualitative study of two VR horticultural scenes for relaxation
Source: Front Psychol. 2026 Oct 5;17:1932971. doi: 10.3389/fpsyg.2026.1932971 (PMC13678666; doi:10.3389/fpsyg.2026.1932971)
Supplement: Supplementary file 1 [file Data_Sheet_1.pdf]

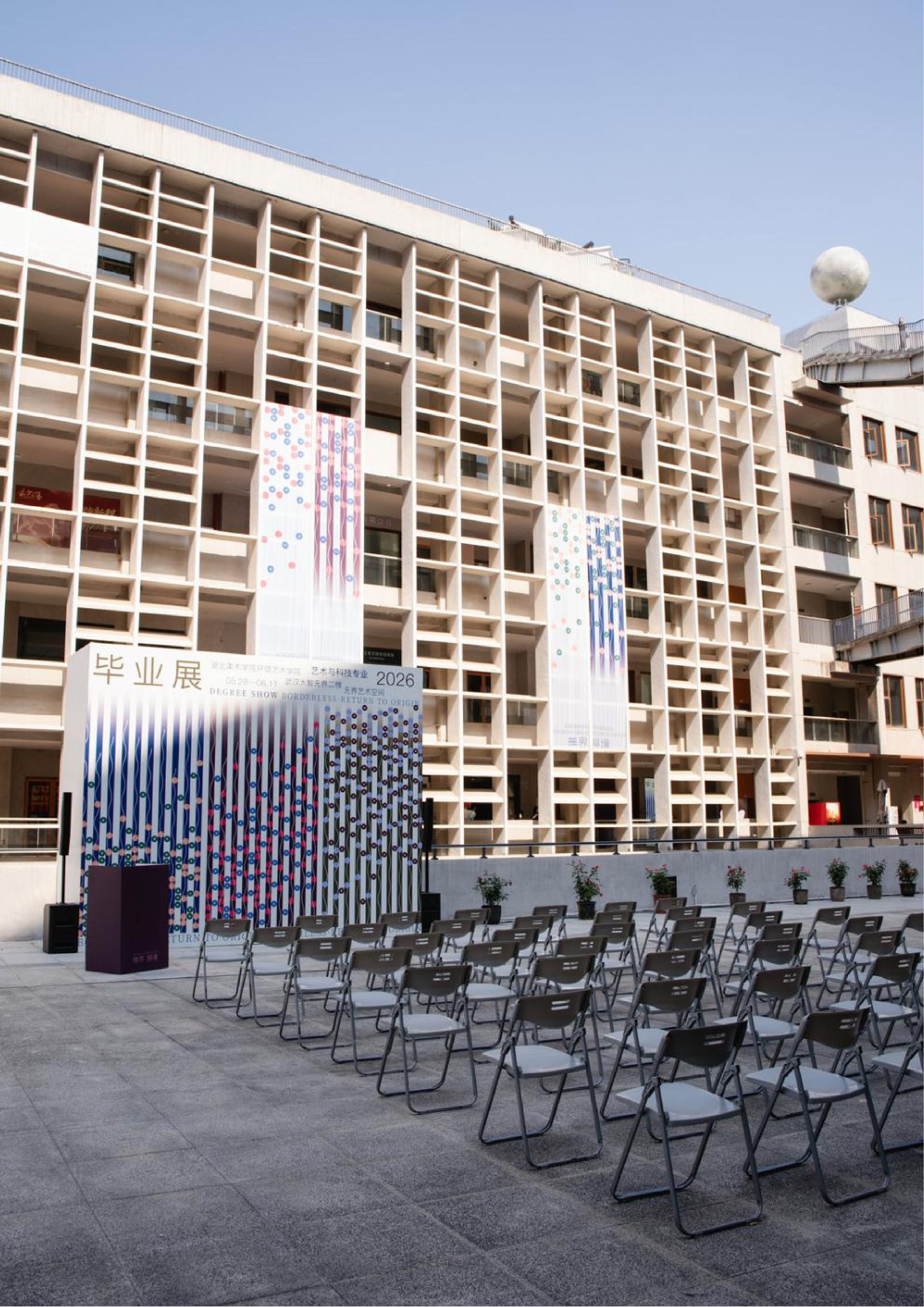
毕业展
艺术与科技专业
2026
DEGREE SHOW BORDERLESS RETURN TO ORIGIN
无界艺术空间

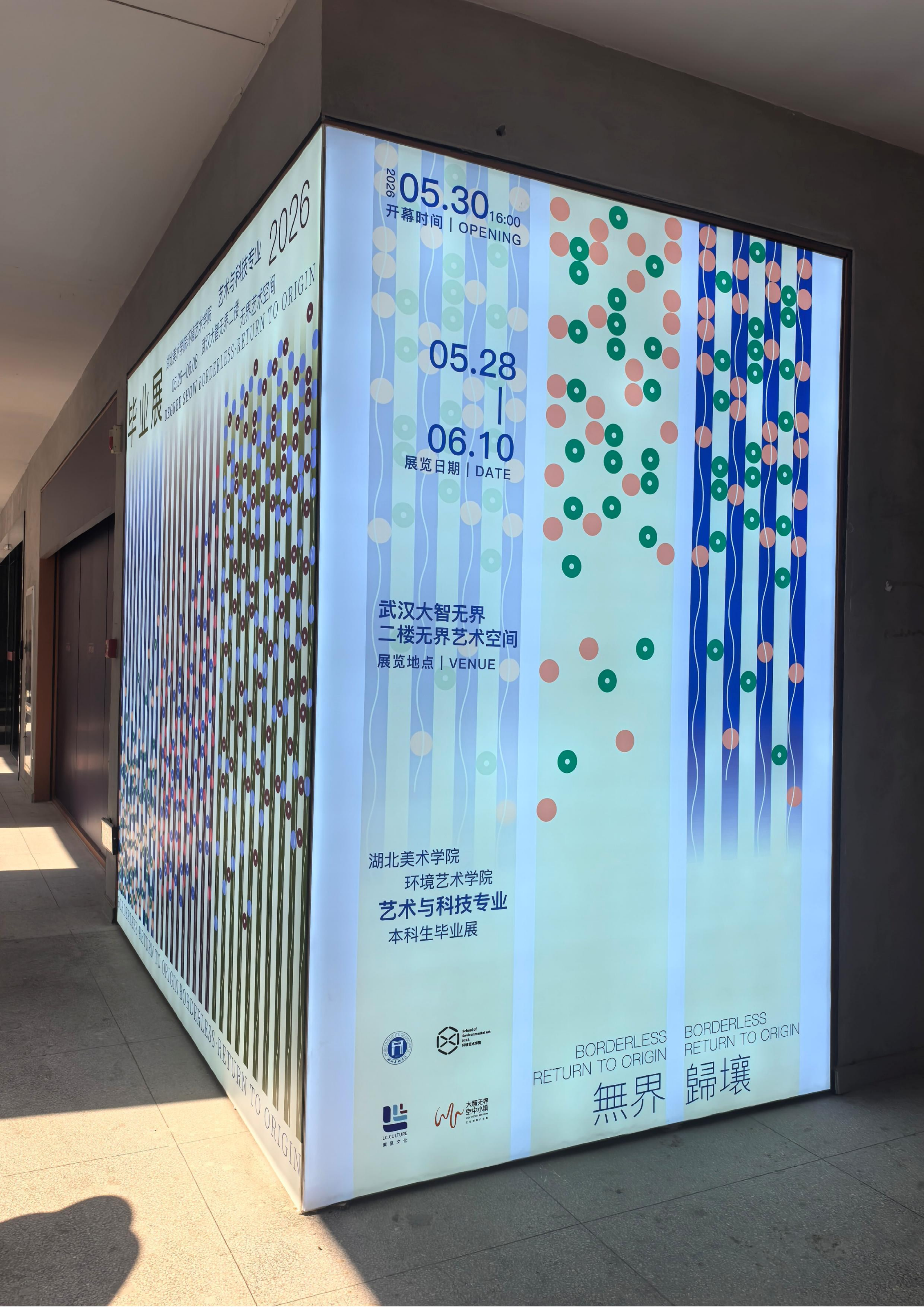

毕业展
湖北美术学院环境艺术学院
艺术与科技专业
2026
05.26—06.08
武汉大智无界二楼
无界艺术空间
DEGREE SHOW BORDERLESS·RETURN TO ORIGIN
2026
05.30 16:00
开幕时间 | OPENING
05.28
|
06.10
展览日期 | DATE
武汉大智无界
二楼无界艺术空间
展览地点 | VENUE
湖北美术学院
环境艺术学院
艺术与科技专业
本科生毕业展
BORDERLESS
RETURN TO ORIGIN
無界
BORDERLESS
RETURN TO ORIGIN
歸壤
BORDERLESS·RETURN TO ORIGIN BORDERLESS·RETURN TO ORIGIN

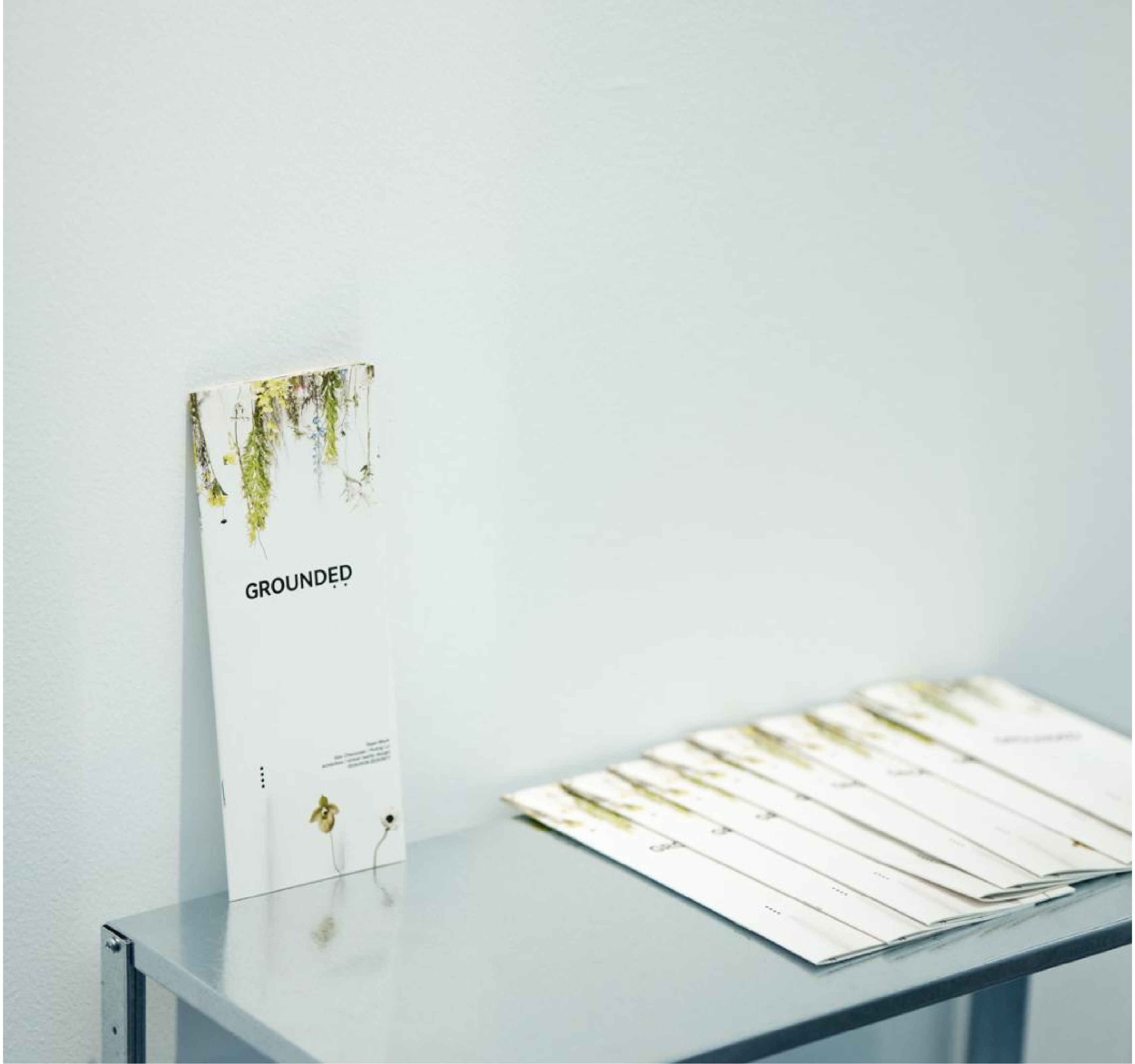
GROUNDED

“我”与土地

ROOTS, SOIL & ME

GROUNDED

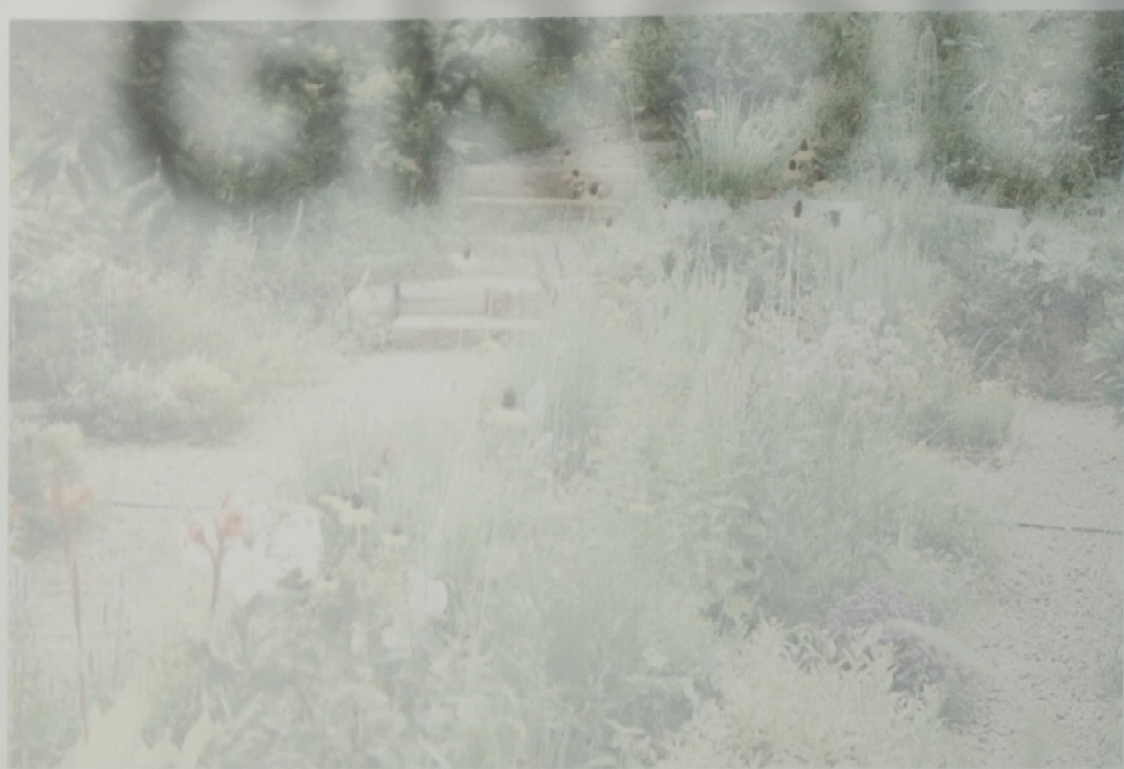

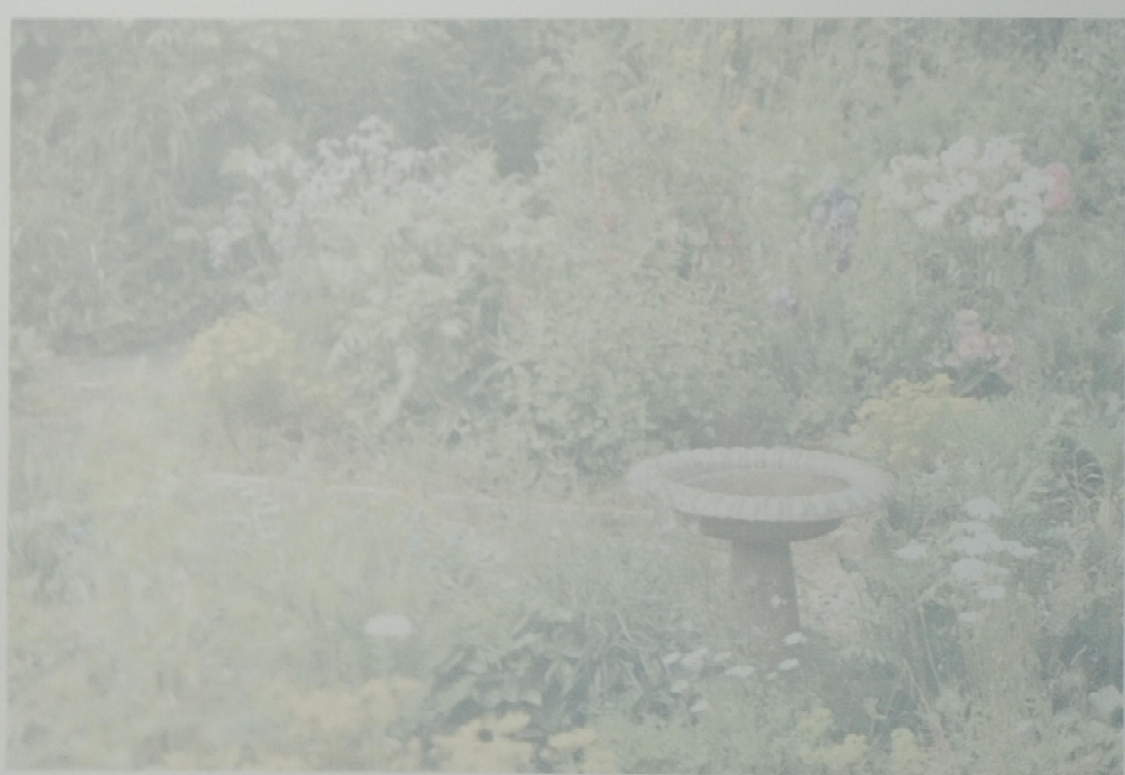

到了大学，我开始真正接触土地，触摸土壤。不再是隔着玻璃观察植物的生长，而是用手指嵌入泥土，感受它的湿度、温度与颗粒的质地。土壤不再只是植物的容器，而成为一种可以被身体感知的存在。

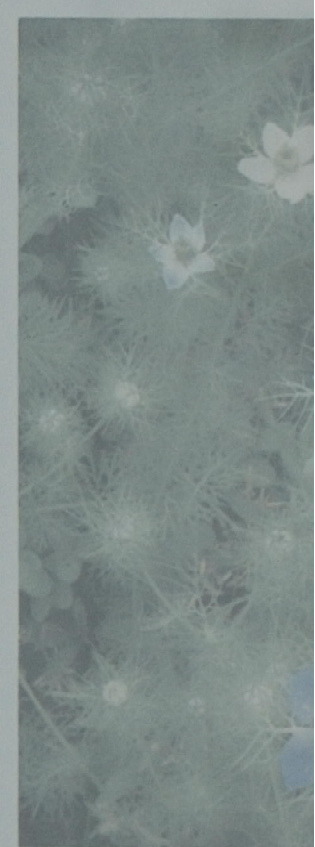

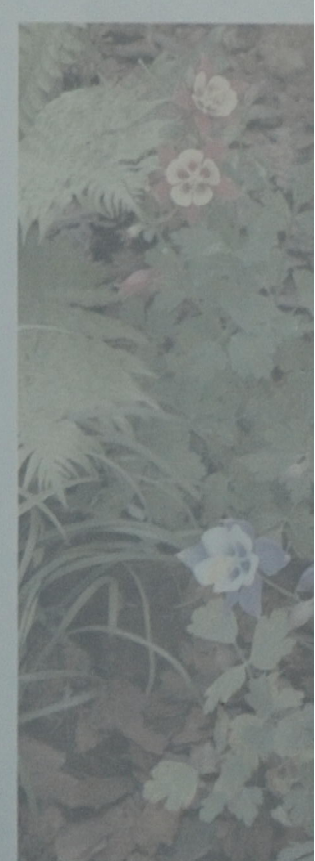

When I entered university, I began to truly engage with the land—to touch the soil itself. No longer observing plant growth through glass, I pressed my fingers into the earth, sensing its moisture, temperature, and granular texture. Soil was no longer merely a

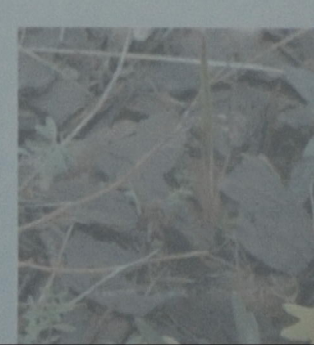

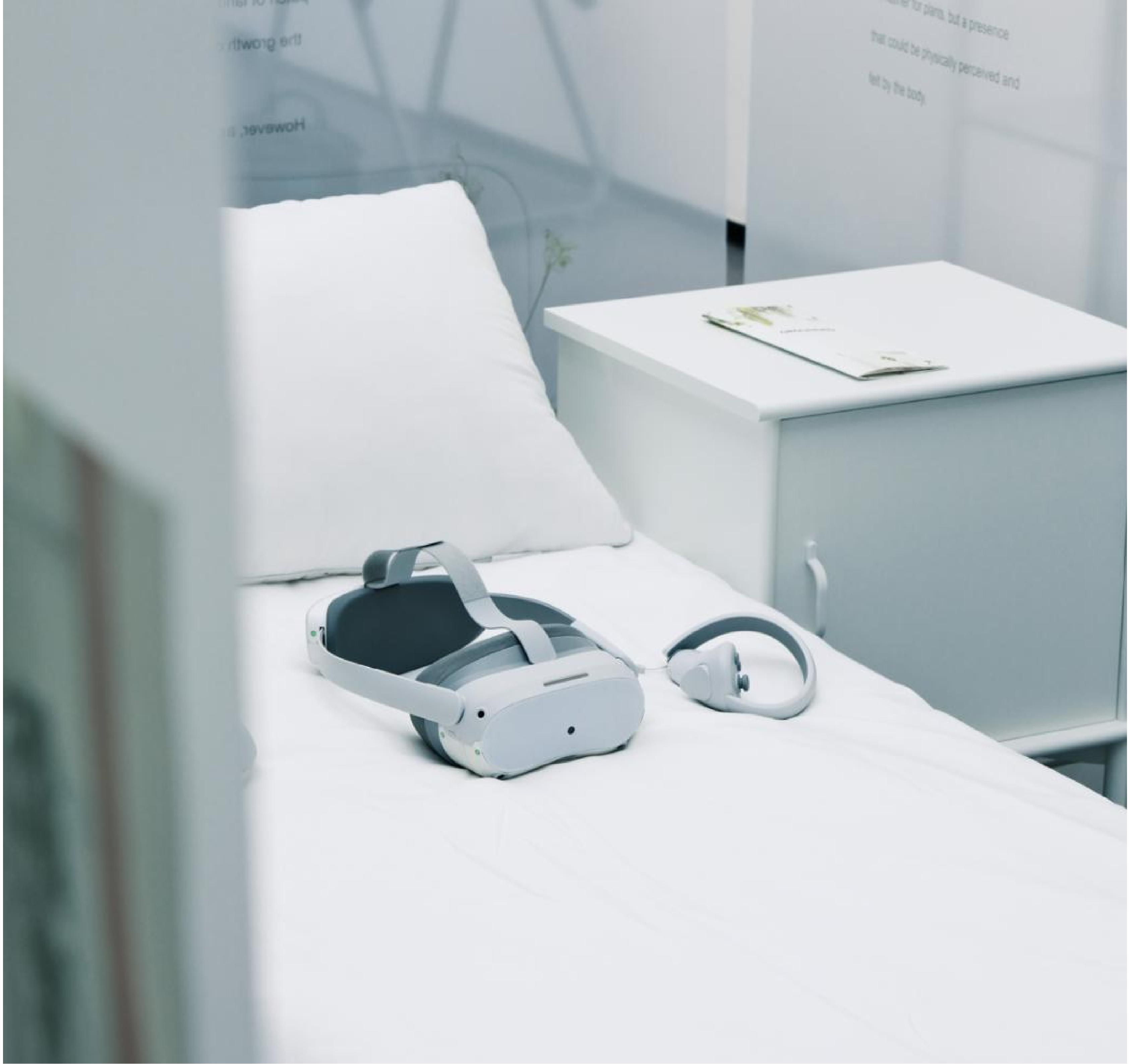
that could be physically perceived and
felt by the body.

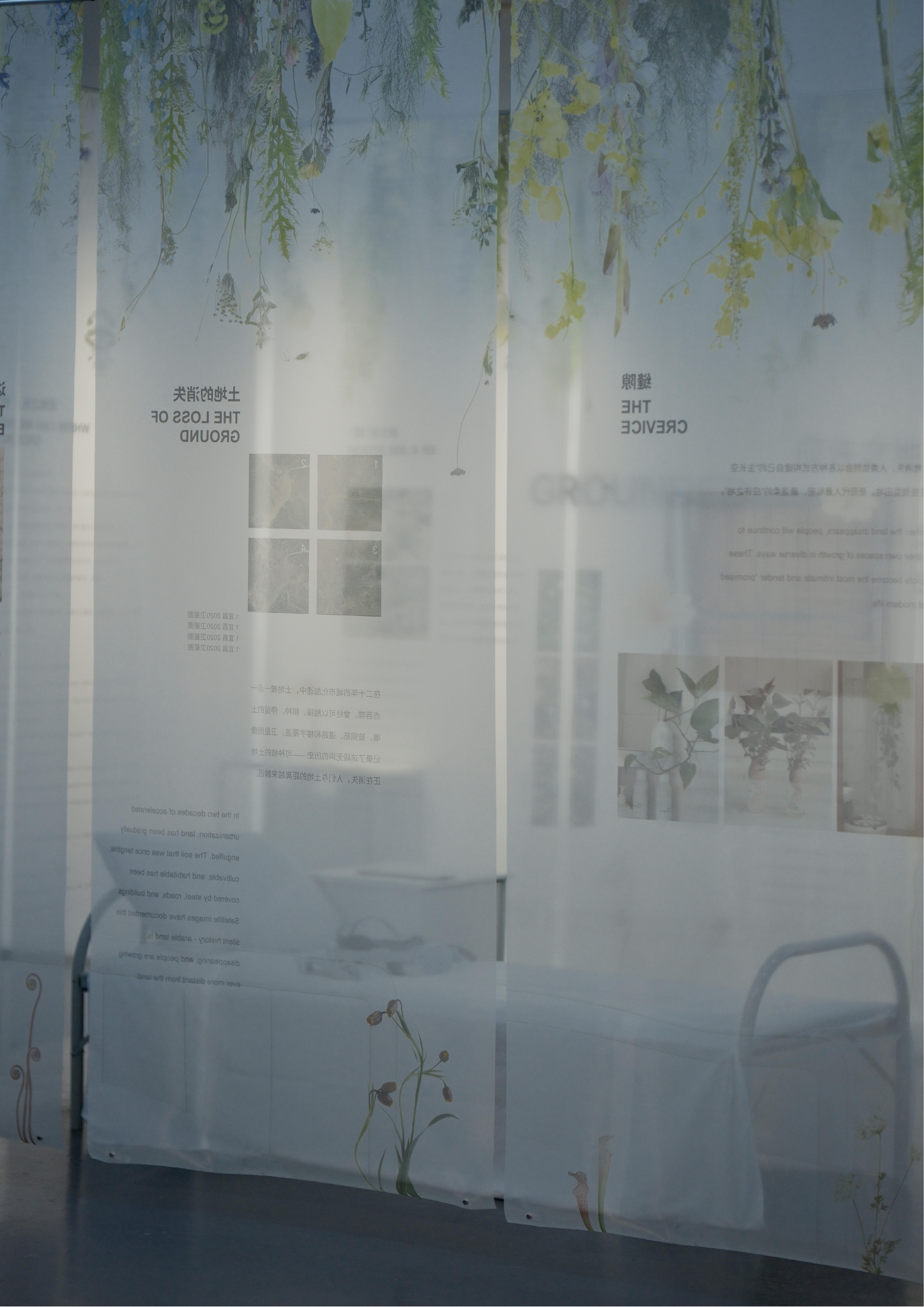
土地的消失
THE LOSS OF GROUND
1 宜昌 2020卫星图
1 宜昌 2020卫星图
1 宜昌 2020卫星图
1 宜昌 2020卫星图
在二十年的城市化加速中，土地被一点一点吞噬。曾经可以触摸、耕种、停留的土壤，被钢筋、道路和楼宇覆盖。卫星图像记录了这段无声的历史——可种植的土地正在消失，人们与土地的距离越来越远。
In the two decades of accelerated urbanization, land has been gradually engulfed. The soil that was once tangible, cultivable, and habitable has been covered by steel, roads, and buildings. Satellite images have documented this silent history - arable land is disappearing, and people are growing ever more distant from the land.
缝隙
THE CREVICE

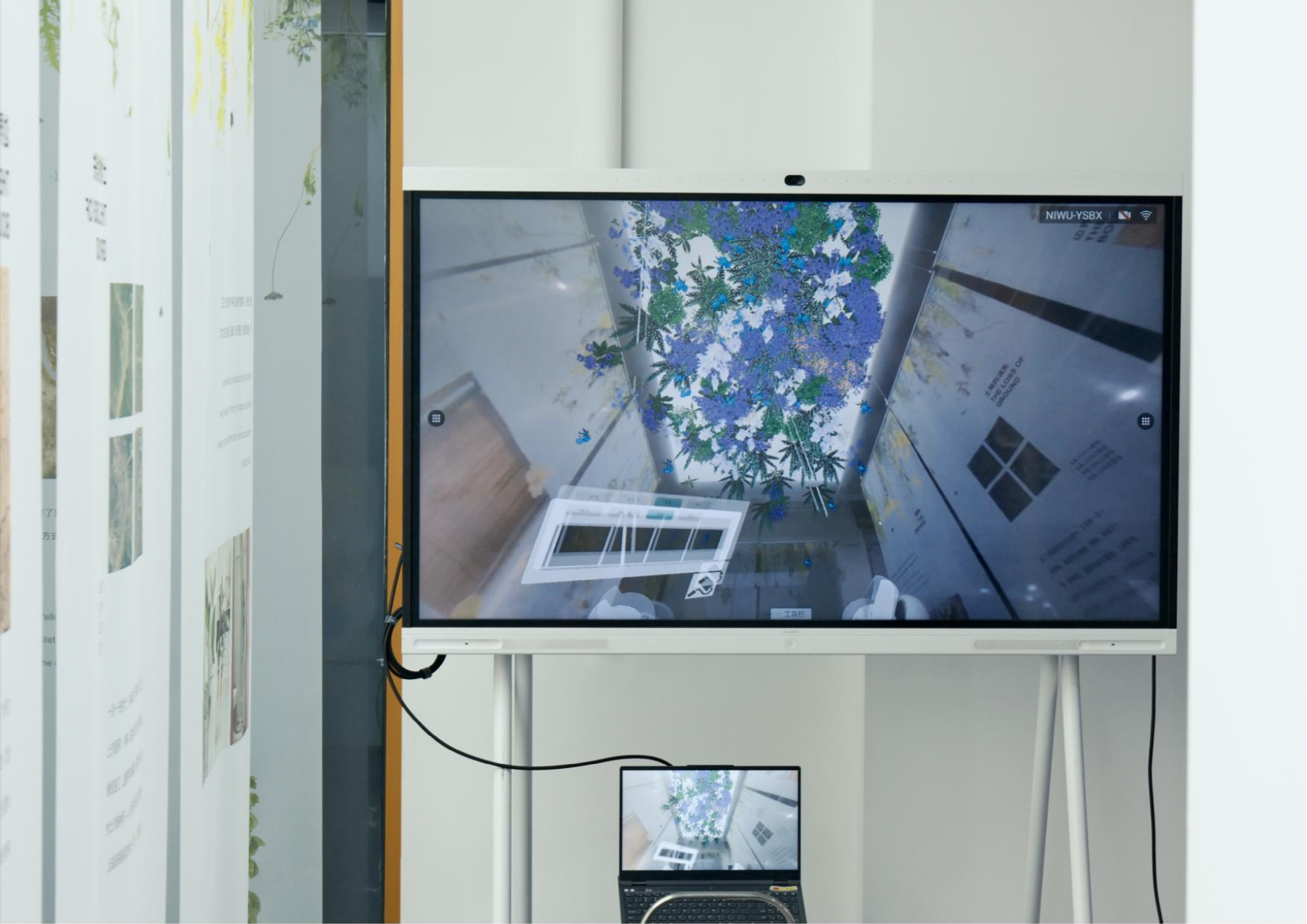
NIWU-YSBX
THE LOSS OF GROUND

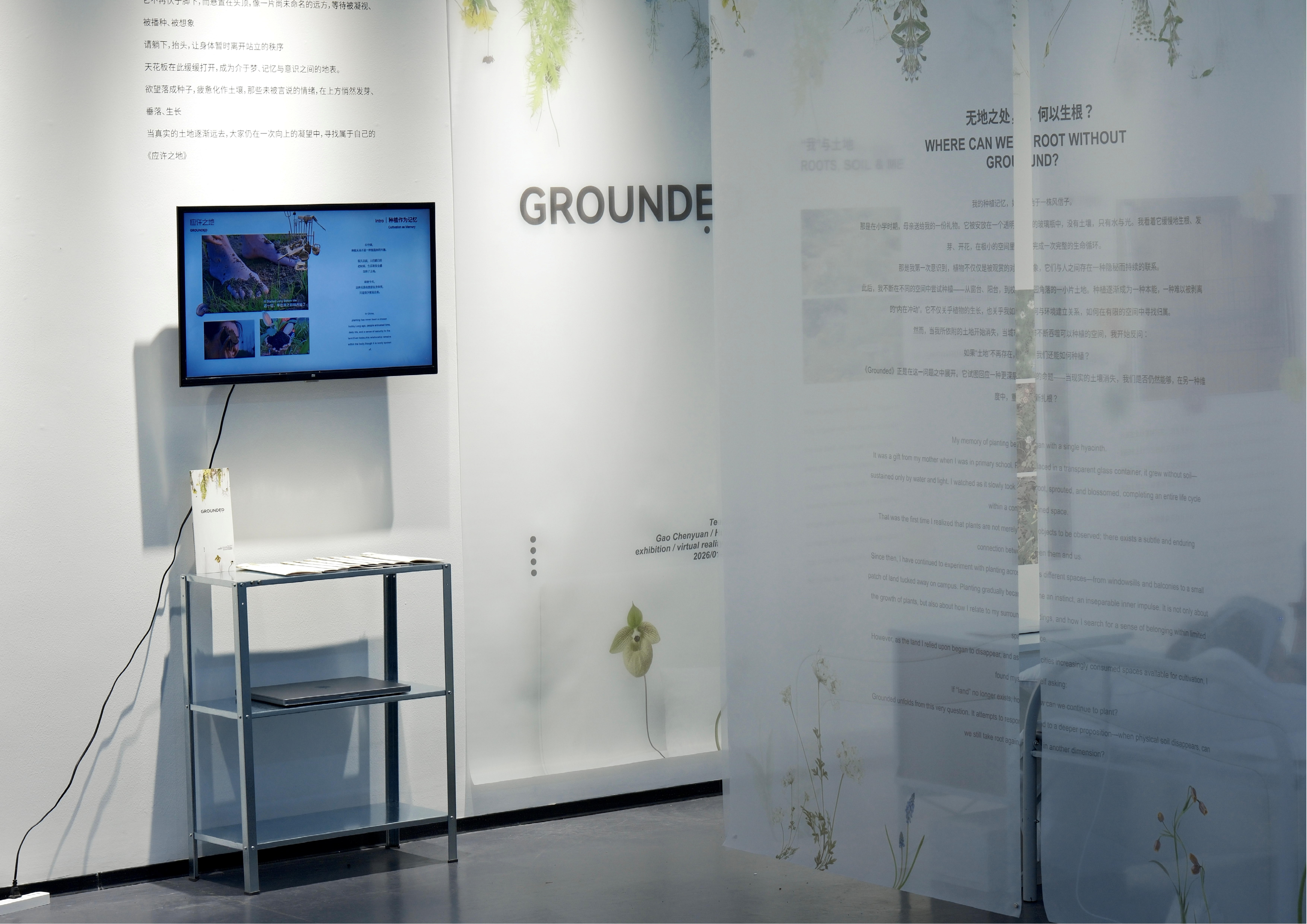
已不再伏于脚下，而悬直在头顶，像一片尚未命名的远方，等待被凝视、
被播种、被想象
请躺下，抬头，让身体暂时离开站立的秩序
天花板在此缓缓打开，成为介于梦、记忆与意识之间的地表。
欲望落成种子，疲惫化作土壤，那些未被言说的情绪，在上方悄然发芽、
垂落、生长
当真实的土地逐渐远去，大家仍在一次向上的凝望中，寻找属于自己的
《应许之地》
应许之地
GROUNDED
Intro | 种植作为记忆
Cultivation as Memory
GROUNDED
GROUNDE
无地之处，
何以生根？
如果"土地"不再存在，
我们还能如何种植？
My memory of planting be
gan with a single hyacinth.
If "land" no longer exists, h
w can we continue to plant?
