# Supplementary material for: Luminous vs. nocturnal canopy: a comparative qualitative study of two VR horticultural scenes for relaxation
Source: Front Psychol. 2026 Oct 5;17:1932971. doi: 10.3389/fpsyg.2026.1932971 (PMC13678666; doi:10.3389/fpsyg.2026.1932971)
Supplement: Supplementary file 2 [file Supplementary_file_1.pdf]

Appendix

Appendix A: Questionnaire (Template)

VR Horticultural Therapy Scene Experience: Background Information Questionnaire

Participant Code: P____ Date:________

Experience Order: □ Luminous Canopy first, then Nocturnal Canopy / □ Nocturnal Canopy first, then Luminous Canopy

Section 1: Basic Information

1. Your age: ____

2. Your gender:

□ Male □ Female □ Other

Section 2: VR Experience

3. Have you used virtual reality (VR) devices before?

□ Yes (please answer Q4)

□ No (please skip to Q5)

4. If yes, approximately how many times?

□ 1–2 times

□ 3–5 times

□ 6 or more times

Section 3: Daily Nature Preferences

5. How often do you usually spend time in natural environments? (e.g., park walks, botanical gardens, gardening)

□ Rarely (less than once a month) □ 1–2 times a month

□ 1–2 times a week □ Almost daily

6. Do you generally prefer to go out during the daytime or at night?

□ Daytime

□ Nighttime

□ No clear preference

Researcher's Notes (recto-verso)

Post-Experience Record:

Relaxation rating for Scene 1 (1–10): ____

Relaxation rating for Scene 2 (1–10): ____

Preference: □ Luminous Canopy / □ Nocturnal Canopy / □ No preference

Preference intensity (1–10): ____

Post-experience comments:

Researcher's Signature: ________

Appendix B: Field Notes (N = 14)

Study: User Experience Study of VR therapeutic environments

Researchers: Zhang Fan, Gao Chenyuan, Huang Lu

Testing Period: 30 May 2026 – 9 June 2026

Testing Location: Wujie Art Space, Dazhi Wujie, Wuhan, China

Participant Code	Gender	Age	Scene	Key Observation / Participant Feedback	Theme Category
P01	Female	58	Luminous Canopy	Seeing the daisies and dandelions reminded me of running through the fields when I was a child.	ER, FR
			Nocturnal Canopy	The dark one slowed down my whole mind, very quiet.	ER
P02	Male	45	Luminous Canopy	The purple of the lavender was very calming, the color was comfortable	ER, FR
			Nocturnal Canopy	The dark mushrooms are very special, you could never see them in reality, but they felt so peaceful	ER, SE
P03	Female	35	Luminous Canopy	The bird calls from the left made me feel like birds were right beside me, and my mood lifted immediately.	ER
			Nocturnal Canopy	The wind chimes from afar made me feel like I was standing in a vast space.	ER
P04	Female	30	Luminous Canopy	Bright color is good and gives me good mood.	SE
			Nocturnal Canopy	A bit uncomfortable at first, but after getting used to it, it was very peaceful.	SE
P05	Male	28	Luminous	This is like the flowers my father planted when	FR, SE

Participant Code	Gender	Age	Scene	Key Observation / Participant Feedback	Theme Category
			Canopy	I was a child, very familiar	
			Nocturnal Canopy	The darkness has more mystery	SE, CI
P06	Female	26	Luminous Canopy	The moment I stepped in, my spirits lifted — I almost felt like I could smell flowers.	ER
			Nocturnal Canopy	I was already quite tired today and just wanted a quiet place to stay.	CI
P07	Female	25	Luminous Canopy	I'm in a good mood today, I want to walk in the sunshine.	CI
			Nocturnal Canopy	I don’t have any strong feelings just feel dark plants are a little odd	ER
P08	Male	24	Luminous Canopy	I recognize daisies and lavender, they are familiar things, and that feels comforting.	FR, SE
			Nocturnal Canopy	Is that purple blue mushroom? Never seen that before.	FR, SE
P09	Female	22	Luminous Canopy	Things I recognize make me more relaxed; plants that are too strange actually make me a bit nervous.	FR
			Nocturnal Canopy	Precisely because I don't recognize them, they feel special, like being in another world	FR
P10	Female	20	Luminous Canopy	The blooming flowers made me feel like everything still has hope.	ER
			Nocturnal Canopy	The deep purple of the dark orchid made me not want to speak, just quietly look at it.	ER

Participant Code	Gender	Age	Scene	Key Observation / Participant Feedback	Theme Category
P11	Female	18	Luminous Canopy	So that's how flowers bloom — I never really paid much attention before	ER
			Nocturnal Canopy	I would like to choose different ones depending on my mood, it's not fixed.	CI
P12	Male	35	Luminous Canopy	I usually get up early and walk in the park in the weekends, so the bright one suits me better	CI
			Nocturnal Canopy	I don't really like night time, so I don't like the dark version.	CI
P13	Male	6	Luminous Canopy	Blooming is so interesting! And the music is lovely too!	ER
			Nocturnal Canopy	I like bright colors, and I like this dark one too	ER, SE
P14	Male	42	Luminous Canopy	I feel bright as soon as I see the flower blooming.	ER
			Nocturnal Canopy	Dark colors kind of bring my mood down.	ER, SE

Theme Categories Reference:

ER = Distinct Emotional Response Patterns
FR = Plant Familiarity and Recognition
SE = Safety and Exploration
CI = Contextual Fit and Individual Differences

* Field notes were recorded by Huang Lu (P01, P03, P05, P06, P08–P14) and Gao Chenyuan (P02, P04, P07).

Appendix C: Ethical Review (English version)

Ethical Review Opinion of the School of Environmental Art, Hubei Institute of Fine Arts

Review Number: SEA2602

I. Basic Project Information

Project Title: User Experience Study of Art Therapy Scenes Based on Virtual Reality Technology

Project Leader: Zhang Fan, +8618*****3443

Affiliation: School of Environmental Art, Hubei Institute of Fine Arts

Research Type: Human behavioral and psychological experience research

Testing Period: 30 May 2026 - 8 June 2026

Testing Location: Wujie Art Space, Dazhi Wujie, Wuhan, China

II. Research Overview

This study employs virtual reality (VR) technology to construct two art therapy scenes with distinct visual tones, aiming to explore the influence of environmental visual style on user experience and emotional responses. Voluntary participants from the general population will be recruited (planned sample size within 30, age below 70) for short immersive VR experiences (approximately 3 to 5 minutes per session). Following the experience, subjective feedback, scene preferences, and emotional response data will be collected through questionnaires and on-site observational records. The study does not involve the collection of physiological indicators, biological sample testing, pharmacological interventions, or any invasive procedures.

III. Ethical Review Opinion

In accordance with the Measures for the Ethical Review of Science and Technology (Trial) (GuoKeFaJian [2023] No.167), the Measures for the Ethical Review of Life Sciences and Medical Research Involving Humans (Order No. 4 of the National Health Commission and Three Other Departments), and the Constitution of the Academic Committee of the School of Environmental Art, Hubei Institute of Fine Arts, the committee has conducted the following review:

1. Informed Consent

Researchers are required to fully inform all participants (and their legal guardians in the case of minors) prior to the experience regarding the research purpose, procedures, data usage, and the right to withdraw voluntarily, and to obtain written informed consent. The informed consent process must be conducted properly.

2. Risk and Benefit Assessment

This study involves only short-term VR immersion and questionnaire administration, without any physiological intervention, pharmacological use, or invasive procedures, and poses no foreseeable harm to participants' physical or mental health. Mild dizziness that may occur during VR exposure

has been effectively mitigated in the study design through session duration limits (3–5 minutes) and rest intervals. The research presents low risk with clear scientific and design implications, and the risk-benefit ratio is considered reasonable.

3. Privacy and Data Protection

Researchers are required to anonymize all personal information and feedback data. All data shall be used exclusively for academic research purposes, shall not involve video or audio recordings, and shall not disclose participants' identities. Data storage must comply with confidentiality requirements.

4. Protection of Vulnerable Populations

This study may involve minor participants (under 18 years of age). Written informed consent must be obtained from their legal guardians, and minors must be accompanied by a guardian or researcher throughout the experience. Relevant procedures shall comply with ethical requirements for the protection of vulnerable populations.

IV. Review Conclusion

This study meets the ethical standards for science and technology and is approved to proceed as proposed. Any adverse events related to the research or major changes to the protocol that occur during the study shall be promptly reported to this committee.

School of Environmental Art, Hubei Institute of Fine Arts (Seal)

Date of Review: 22 May 2026

Note: The actual testing period was extended by one day (to June 9, 2026) beyond the initially approved period (May 30 – June 8, 2026), as the actual exhibition ended on June 10, 2026.

Appendix D: Informed Consent Form (Template)

Informed Consent Form

Study Title: Luminous vs Nocturnal Canopy: A Comparative Qualitative Study of Light and Dark VR Horticultural Therapy Scenes for Stress Relief

Research Institution: Hubei Institute of Fine Arts

Researchers: Zhang Fan, Gao Chenyuan, Huang Lu

Contact: +86 18*****3443

1. Study Purpose

This study aims to explore the influence of different visual tones in virtual reality (VR) horticultural scenes on user experience. You are invited to experience two VR scenes—Luminous Canopy (light-colored plants) and Nocturnal Canopy (dark-colored plants)—and share your experience.

2. Participation Content

If you agree to participate, you will:

1). Complete a brief background information questionnaire (approximately 1 minute)

2). Wear a Pico 4 VR headset, lie in bed, and experience two VR scenes (approximately 3–5 minutes each)

3). Rate your relaxation level on a 1-to-10 scale after each scene

4). Indicate your scene preference and rate your preference intensity after both scenes

5). Participate in a brief 1–3 minute follow-up interview to share your experience

Total duration: Approximately 10–15 minutes

3. Potential Risks and Discomfort

This VR experience is a low-risk activity. Some users may experience mild dizziness or visual fatigue during VR exposure; you may pause or withdraw at any time if you feel discomfort. If you experience any discomfort during the experience, please inform the researcher immediately.

4. Privacy and Confidentiality

All collected information will be strictly confidential:

- Your name will not appear in any research report or publication; all data will be identified by anonymous codes

- No video or audio recordings will be made; the researcher will only document your verbal feedback in writing

- All data will be stored on a password-protected device accessible only to the research team

- After the study is completed, all personally identifiable information will be destroyed

5. Voluntary Participation and Withdrawal

Participation is entirely voluntary. You have the right to withdraw at any time without providing a reason and without any negative consequences. If you feel discomfort during the VR experience, you also have the right to stop immediately.

6. Participant's Statement

I have read and understood the above information. I have been informed of the nature, purpose, participation content, and potential risks of this study. I have had the opportunity to ask questions and received satisfactory answers.

I agree to participate in this study.

Participant's Signature: ________________ Date: ________

7. Researcher's Statement

I have fully explained the purpose, procedures, and precautions of this study to the participant.

Researcher's Signature: ________________ Date: ________
